# Thymalfasin therapy accelerates COVID-19 pneumonia rehabilitation through anti-inflammatory mechanisms

**DOI:** 10.1186/s41479-023-00116-6

**Published:** 2023-09-25

**Authors:** Zirui Wang, Cong Wang, Xiaohua Fei, Haixing Wu, Peiqin Niu, Changxing Shen

**Affiliations:** 1 Department of Respiratory and Critical Medicine, the Fifth People’s Hospital of Wujiang District, Suzhou, 215211 JS China; 2 Information Centre, the Fifth People’s Hospital of Wujiang District, Suzhou, 215211 JS China; 3 Department of Pharmacy, the Fifth People’s Hospital of Wujiang District, Suzhou, 215211 JS China; 4 Department of Medical Record Statistics, Shanghai Tenth People’s Hospital, Tongji University School of Medicine, Shanghai, 200072 China; 5 Department of Integrated Traditional Chinese and Western Medicine, Shanghai Tenth People’s Hospital, Tongji University School of Medicine, 200072 Shanghai, China

**Keywords:** Thymalfasin, Common COVID-19, Severe pneumonia, Inflammation, Timing of treatment

## Abstract

**Introduction:**

Thymosin drugs are commonly used for the treatment of viral infections due to their immunomodulatory effects. The comprehensive clinical efficacy of Thymalfasin therapy for COVID-19 associated pneumonia is not yet fully researched, another issue, whether the use of thymosin drugs can reduce the rate of COVID-19 progression to severe pneumonia has not been well documented. The aim of the present study was to multi-angle evaluate the clinical efficacy of Thymalfasin therapy for COVID-19 pneumonia by retrospective review of the clinical data of 338 inpatients with common COVID-19 infection who received treatment in our hospital.

**Methods:**

The primary index of observation was whether progression to severe pneumonia occurred within a week after admission, and the secondary indexes were the length of hospital stay, time of negative conversion of COVID-19 antigen, the number of peripheral lymphocytes and white blood cells (WBC), and C-reactive protein (CRP) and procalcitonin (PCT) levels,and the control of pneumonia related symptoms, for example, fever, listlessness, inflammatory exudate area shown on lung CT (%).

**Results:**

The length of hospital stay of patients in Thymalfasin group was significantly shorter than that of patients in the control group (*p* < 0.01). The proportion of relief of pneumonia related symptoms (fever, fatigue) in the Thymalfasin therapy group was significantly higher than that in the control group, and the inflammatory exudate area shown on CT was significantly lower than that in the control group (*p* < 0.05). Multivariate logistic regression analysis showed that the use of Thymalfasin was an independent protective factor affecting the progression to severe pneumonia. Multifactorial Cox model analysis indicated that negative conversion of COVID-19 antigen was significantly faster in patients using Thymalfasin and younger patients.

**Conclusion:**

Thymalfasin therapy has shown excellent clinical efficacy in the treatment of COVID-19 pneumonia, it can reduce inflammatory reactions, promote the relief of COVID-19 pneumonia related symptoms such as fever and fatigue, facilitate effusion absorption, and accelerate COVID-19 pneumonia recovery. Thymalfasin can prevent progression of common COVID-19 infection to severe pneumonia via multiple immunity-enhancing and anti-inflammatory protective mechanisms.

## Introduction

COVID-19 has posed a great threat to the health of the globe population, including many patients who died due to progression to severe pneumonia. The human powerful anti-viral immune system is an important foundation against viral invasion to realize rehabilitation. Lymphocytes, including B lymphocytes, T lymphocytes and natural killer cells (NK), are frontline “soldiers” against exogenous viral infections. Viral stimulation induces B lymphocytes to produce antibodies to differentiate and neutralize the invading virus, which forms the first line of defense against viruses. Infected cells are subsequently killed by T lymphocytes. Lymphocytes play an primarily important role in the fight against viral infection. It is therefore very important to increase the number of T cells and their anti-viral function in COVID-19 patients for the sake of promoting successful rehabilitation [1]. However, most COVID-19 patients, especially elderly and critically ill patients, often present severe lymphocytopenia [2]. Thymalfasin, also known as thymosin alpha-1, is a polypeptide hormone generated by thymic epithelial cells which can effectively increase the number of T cells, support T cell differentiation and maturation, and reduce apoptosis. Pathological observation of previous clinical studies [3] have confirmed the therapeutic efficacy of Thymalfasin in patients infected by hepatitis B virus (HBV), hepatitis C virus (HCV) and human immunodeficiency virus (HIV).

Some current studies have demonstrated that thymosin drugs can increase the number of lymphocytes of COVID-19 patients and even reduce the mortality rate of critically ill patients. However, there are also other studies questioning their therapeutic efficacy [4, 5], and therefore larger numbers of clinical studies are still required to confirm whether they can reduce the rate of progression to severe illness, especially in patients with the moderate form of COVID-19 infection. The aim of the present study was to multi-angle evaluate the clinical efficacy of Thymalfasin therapy for COVID-19 pneumonia by retrospective review of the clinical data of 338 inpatients with common COVID-19 infection who received treatment in the Fifth People’s Hospital of Wujiang District (Suzhou, China) between December 2022 and January 2023.

## Patients and methods

### Diagnosis and grouping

**Fig. 1:**
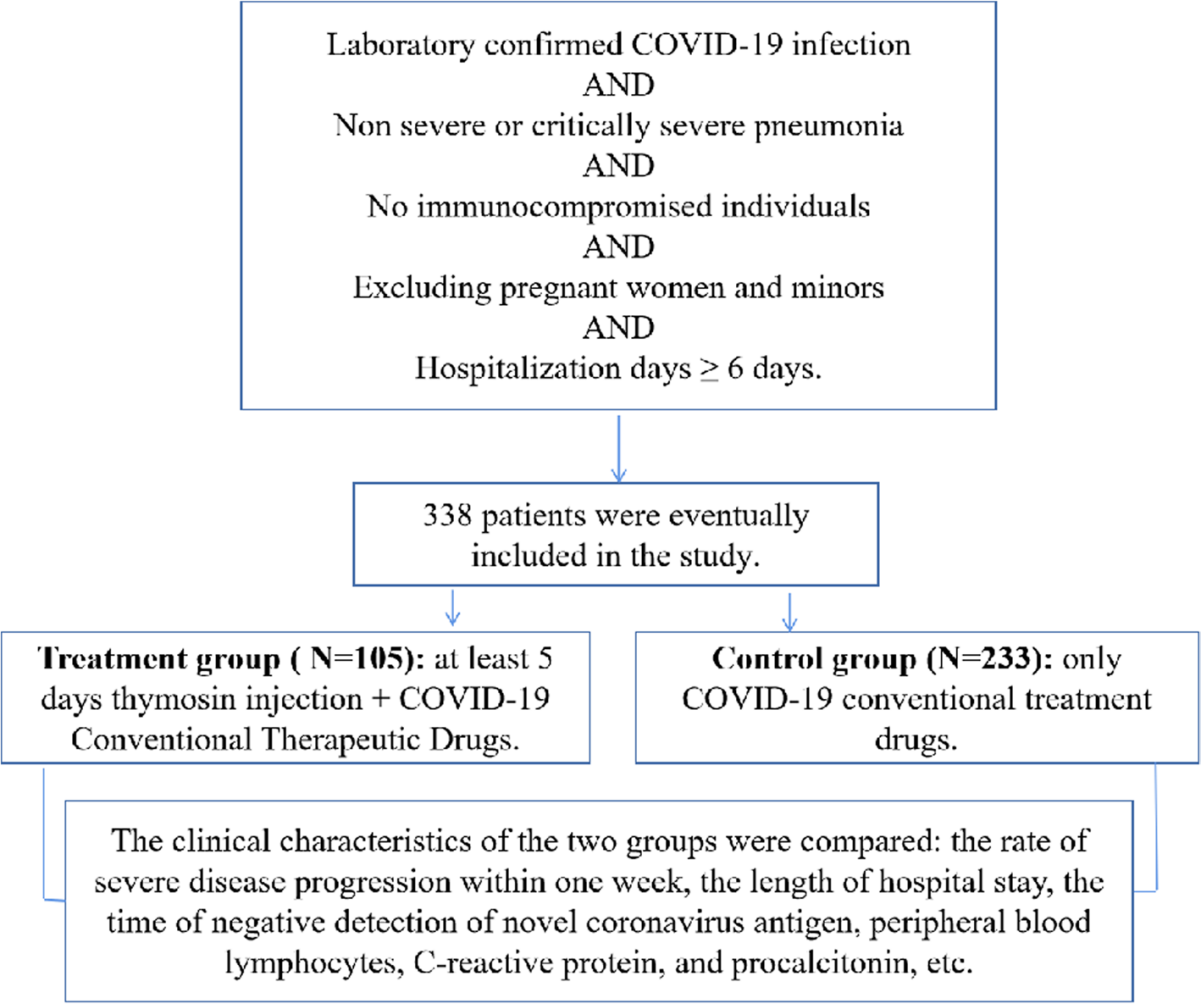
The flow-process diagram of the study

 Medical data of patients aged 28–105 years with non-severe COVID-19 infection and received treatment at the department of internal medicine of the Fifth People’s Hospital between December 2022 and January 2023 were reviewed retrospectively. The diagnosis and clinical typing of these patients were in accordance with the Diagnosis and Treatment Program for Novel Coronavirus Infection (Tenth Edition on Trial) issued by China’s National Health Commission. Patients who met with one of the following conditions were diagnosed as having severe pneumonia: shortness of breath at a rate of RR ≥ 30 breaths/min; digital oxygen saturation ≤ 93% upon inspiration at rest; PaO2/FiO2 ≤ 300mmHg (1mmHg = 0.133 kPa) [In high-altitude (> 1000 m) areas], PaO2/FiO2 should be adjusted by using the following equation: PaO2/FiO2×[760/atmospheric pressure (mmHg)];progressive exacerbation of the clinical symptoms; and lung imaging showing significant progression of the lesion greater than 50% within 24~48 hours. Patients with non-severe COVID-19 who had been hospitalized for at least 6 days and adult patients with non-severe COVID-19 were finally included for analysis. The use of Thymalfasin was voluntary. Part of the patients were unable to use this drug because of the financial problem were included as the control group, and patients who could financially afford the use of treatment with Thymalfasin for more than 5 days were included as the Thymalfasin treatment group. The dosage and administration of Thymalfasin Injection were 1.6 mg/via for subcutaneous injection, QD. Patients in both groups also received COVID-19 routine treatment as the basic treatment, including Methylprednisolone for anti-inflammation and Ambroxol for reducing phlegm and relieving cough, in addition to other supportive and symptomatic therapies. The details of the inclusion criteria are listed in Fig. 1. The primary index of observation was whether progression to severe pneumonia occurred within a week after admission, and the secondary indexes were the length of hospital stay, time of negative conversion of COVID-19 antigen, the number of peripheral lymphocytes and white blood cells (WBC), and C-reactive protein (CRP) and procalcitonin (PCT) levels. Peripheral WBC and lymphocyte counts, and CRP and PCT levels during hospitalization were compared between the two groups. The length of hospital stay, the rate of progression to severe pneumonia or critical pneumonia, and the time of negative conversion of COVID-19 throughout the admission period were assessed in both groups to judge the clinical therapeutic efficacy of Thymalfasin.

### Application and management of Thymalfasin

Patients in Thymalfasin group received a subcutaneous injection of Thymalfasin daily for at least 5 consecutive days. After re-examining the relevant indexes, Thymalfasin treatment was continued until the condition turned for the better and the patient was discharged, or until the condition progressed to severe or critical pneumonia. Before use, each vial of Thymalfasin (1.6 mg) was dissolved in 1ml injection solution for subcutaneous injection rather for intramuscular or intravenous injection.

### Data collection

The clinical records, nursing records, laboratory test results and chest X-ray radiograms or CT images of all included patients were collected and handled in the specially designed data collection forms by two researchers to ensure the accuracy of the data.

## Results

No patient in our series was complicated by severe cardiac, hepatic or renal dysfunction, nor had autoimmune disease. There were no pregnant women or juveniles. According to these criteria, 105 patients were in Thymalfasin group, and 233 patients were in the control group. There were no significant differences in sex and age between the two groups. However, the number of peripheral WBC and lymphocytes in Thymalfasin group was significantly lower than that in the control group (*p* < 0.01). The level of CRP in Thymalfasin group was insignificantly higher than that in the control group. There was no significant difference in PCT level between the two groups. The details are listed in Table 1. Re-examination of the laboratory indexes at day 5 after Thymalfasin injection showed that the number of lymphocytes was increased significantly (0.29 ± 0.4 vs. 30.86 ± 0.57, *p* < 0.01) and CRP level was decreased significantly (-57.9 ± 38.82 vs. -42.2 ± 48.44, *p* = 0.0016). In addition, the number of WBC and lymphocytes in Thymalfasin group was significantly higher than that in the control group (*p* < 0.05). The length of hospital stay of patients in Thymalfasin group was significantly shorter than that in the control group (*p* < 0.01). The proportion of relief of pneumonia related symptoms (fever, fatigue) in the Thymalfasin therapy group was significantly higher than that in the control group, and the inflammatory exudate area shown on CT was significantly lower than that in the control group (*p* < 0.05). The rate of progression to severe pneumonia within a week and the time of negative conversion of the first antigen detection in Thymalfasin group were shorter than those in the control group, though the difference was not statistically significant. The details are listed in Table 2. Univariate logistic regress analysis of factors affecting progression to severe pneumonia showed that the use of Thymalfasin was a protective factor, while elevation of WBC, PCT and CRP was a risk factor. The details are listed in Table 3. Multivariate logistic regression showed that the use of Thymalfasin was an independent protective factor of progressing to severe pneumonia, while CRP elevation was an independent risk factor. The details are listed in Table 4. Multivariate Cox model analysis showed that the time of first negative conversion was significantly faster in patients using Thymalfasin and younger patients (Table 5).

**Table 1:** Basic information of the included patients

	0 (*n* = 233)	1 (*n* = 105)	t/χ^2^	*P*
Gender (M/F)	128/105	61/44	0.178	0.67
Age (years)	77.6 ± 12.4	77.0 ± 9.21	0.46	0.64
WBC	6.12 ± 3.07	4.94 ± 2.14	4.07	**< 0.01**
LYM	0.86 ± 0.47	0.58 ± 0.27	6.85	**< 0.01**
CRP	59.8 ± 51.8	70.8 ± 45.69	-1.95	**0.05**
PCT	0.20 ± 0.48	0.20 ± 0.32	0.11	0.91
Fever (yes/no)	(180/53)	(84/21)	0.341	0.88
listlessness(yes/no)	(202/21)	(97/8)	0.21	0.57
Inflammatory exudate area shown on lung CT (%)	34.6 ± 12.3	33.8 ± 14.5	0.59	0.79

*0*: Thymalfasin group，*1: *control group，*WBC* the number of white blood cells and *LYM* lymphocyte: higher in Thymalfasin group than that in control group, *CRP* C-reactive protein: higher in Thymalfasin group than that in control group, *PCT* procalcitonin: no significant difference between the two groups

**Table 2:** Post-treatment status of the patient

	0 (*n *= 233)	1 (*n* = 105)	t/χ^2^	*P*
Treatment days	11.7 ± 4.10	10.4 ± 2.08	4.07	**< 0.01**
Critically ill or not	220/13	104/1	2.82	0.09
Time of negative conversion	9.88 ± 2.82	9.48 ± 2.22	1.40	0.16
WBC	7.14 ± 3.99	6.46 ± 1.65	2.2	**0.02**
WBC change	1.02 ± 4.14	1.52 ± 1.97	-1.51	0.13
LYM	1.15 ± 0.54	1.44 ± 0.55	-4.56	**< 0.01**
LYM change	0.29 ± 0.43	0.86 ± 0.57	-9.16	**< 0.01**
CRP	17.6 ± 20.7	12.8 ± 13.27	2.61	**0.01**
CRP change	-42.2 ± 48.44	-57.9 ± 38.82	3.19	**0.0016**
PCT	0.08 ± 0.19	0.088 ± 0.25	-0.27	0.79
PCT change	-0.12 ± 0.48	-0.11 ± 0.40	-0.243	0.80
Fever (yes/no)	(6/227)	(20/85)	2.78	0.01
listlessness(yes/no)	(19/214)	(35/70)	1.93	0.02
Inflammatory exudate area shown on lung CT (%)	5.8 ± 4.1	14.2 ± 5.6	4.32	0.03

Treatment days: Thymalfasin group shorter than control group, *LYM* lymphocytes: elevated in both groups, and more pronounced in Thymalfasin group, *CRP* C-reactive protein: decreased in both groups, and more pronounced in Thymalfasin group

**Table 3:** Univariate logistic regression analysis of factors affecting progression to severe illness

	OR	Z value	*P*
Use of Thymalfasin or not	0.16	-1.738	**0.08**
Age (65 as the demarcation)	5.661130*10^6^	0.015	0.98
Gender	1.44	0.64	0.52
WBC (10 as the demarcation)	4.45	2.55	**0.01**
CRP (50 as the demarcation)	7.04	2.53	**0.011**
PCT (0.05 as the demarcation)	3.91	1.77	**0.08**
LYM (20% as the demarcation)	1.07	0.13	0.90

Univariate logistic regression showed that the use of Thymalfasin was a protective factor affecting progression to critical illness, while the elevation of WBC, PCT and CRP was a risk factor

**Table 4:** Multivariate logistic regression analysis of factors affecting critical illness progression

	OR	Z value	*P* value
Use of Thymalfasin or not	0.12	-2.019	**0.04**
WBC (10 as the demarcation)	3.11	1.81	0.07
CRP (50 as the demarcation)	8.08	2.66	**0.0078**
PCT (0.05 as the demarcation)	2.66	1.23	0.21

Multivariate logistic regression indicated that the use of Thymalfasin was an independent protective factor of critical illness progression, while CRP elevation was an independent risk factor

**Table 5:** Multivariate Cox model analysis of negative conversion (day)

	HR	Z value	*P* value
Use of Thymalfasin or not	1.40	2.58	**0.01**
Age (65 as the demarcation)	0.23	-7.74	0.00
Gender	1.06	0.58	0.56
WBC (10 as the demarcation)	0.85	-8.2	**0.4**
CRP (50 as the demarcation)	0.88	-1.03	**0.3**
PCT (0.05 as the demarcation)	0.92	-0.69	**0.49**
LYM (20% as the demarcation)	1.02	0.22	0.82

Multivariate Cox model analysis showed that the use of Thymalfasin and younger patients were significant factors for quick negative conversion of COVID-19 antigen

## Discussion

### Thymalfasin therapy accelerates COVID-19 pneumonia rehabilitation through anti-inflammatory mechanisms

Severe or critical COVID-19 pneumonia is the key factor threatening the life of COVID-19 patients, and even those survivors may have adverse health consequences such as pulmonary fibrosis, hypoxia, thrombosis and fatigue, seriously affecting their quality of life. It is therefore necessary to conduct explorative research on the prevention of severe pneumonia. Anti-viral drugs and anti-inflammatory glucocorticoids are routine selections of the clinicians. Although these drugs have definite therapeutic effects in most cases, some cases of common COVID-19 infection would still progress to severe pneumonia. In addition, these therapeutic measures are passive to the human body. More importantly, elderly patients may have thymic tissue degeneration, reduced output of lymphocytes and immune aging due to other metabolic reasons [6]. In addition, lymphocyte exhaustion or even inflammatory storm may occur in elderly patients. For these reasons, researchers have tried to prevent common COVID-19 infection from progressing to severe pneumonia and change the prognosis by enhancing the anti-viral immune function of lymphocytes, activating the anti-viral function of immune cells and regulating the immune response of the human body [7]. Thymosin is a polypeptide hormone that regulates the production of T cells which can promote differentiation of T lymphocytes and enhance their anti-viral activity. It has been repeated applied to treat patients with COVID-19 in recent years. Thymalfasin is a drug with good tolerance. However, the actual therapeutic efficacy of Thymalfasin remains unclear. To fight against viral infection and reduce inflammatory damage, we have tried using Thymalfasin as an adjuvant therapy to treat patients with non-severe COVID-19 infection in our hospital from 2022 to 2023. An overview of the medical records of the therapeutic efficacy of Thymalfasin in 105 subjects in the present study revealed no occurrence of significant Thymalfasin-associated adverse effects. In addition, Thymalfasin was found to have positive therapeutic effects in improving the thymic function as represented by the increased number of lymphocytes and the decreased level of CRP and inflammatory reaction.

Liu et al. [8] reported that thymosin could reverse T cell exhaustion and restore immune reconstruction by promoting thymic output during the severe COVID-19 infection period, especially in COVID-19 patients with the circulating CD8 + T cells or CD4 + T cell count < 400/µL or 650/µL, who could obtain more benefits from thymosin treatment. Our findings are consistent with their results. Some experimental studies [9] revealed that COVID-19 infection induced temporary but severe thymic atrophy and selective exhaustion of CD4+/CD8 + thymocytes, and increased the level of apoptosis in adult BALB/c mice. Therefore, the use of Thymalfasin plays an important role in promoting restoration of the T lymphocyte output function in the thymic tissue. Other studies found that the number of T effector cells was reduced markedly in COVID-19 patients with severe comorbidities [10], suggesting that the decreased number of T cells may be the main reason for the reduced number of lymphocytes and also the reason for the increased severity of COVID-19. The important role of T cells in eradicating viruses highlights the therapeutic significance of thymosin drugs. Yu et al. [11] also reported that thymosin could promote the proliferation of T effector cells in vitro and attenuate lymphocytopenia in COVID-19 patients. Matteucci et al. [12] reported that the expression of genes related to the transduction and expression of cytokine signals was upregulated in COVID-19 patients, and treatment with thymosin drugs reduced the expression of cytokines and inhibited the activation of lymphocytes in vitro. All these findings provide a foundation for the rational use of thymosin drugs in COVID-19, though further clinical studies are required to support the conclusion. Several recent cohort studies [11–13] demonstrated that thymosin could reduce the mortality rate of patients with severe COVID-19 infection by reversing lymphocytopenia and restoring the function of the exhausted T cells. Despite the positive effects of thymosin drugs in the treatment of COVID-19, the sample size of currently available studies is relatively small and therefore clinical studies with larger samples are required to provide more support.

### Thymalfasin is an independent factor for preventing common COVID-19 from progressing to severe pneumonia through multiple protective mechanisms

It was found in our study that the inflammatory level decreased more quickly, and the length of hospital stay was shorter in COVID-19 patients who used Thymalfasin as compared with those in the control group. Multivariate Cox model analysis showed that the use of Thymalfasin promoted negative conversion of the COVID-19 antigen, and that the lime of negative conversion in elderly patients was longer than that in younger patients. The use of Thymalfasin was an independent factor of preventing progression of common COVID-19 to severe pneumonia. All these findings suggest that Thymalfasin can not only promote the T cell output function of the thymic tissue but also enhance anti-viral immunity, alleviate inflammatory damage to the human body and promote rehabilitation. Inflammation-related immune impairment is the pathological foundation of viral infections, and anti-virus, anti-inflammation and organ protection are the important foundation in the treatment of viral infections. Previous studies found that thymosin drugs could inhibit the inflammatory/activated state of monocytes in the treatment of COVID-19 by reducing the release of proinflammatory mediators such as TNF-α, IL-6 and IL-8, and promote the generation of anti-inflammatory cytokines such as IL-10 [14]. Some experimental studies [14, 15] demonstrated that thymosin drugs could regulate actin polymerization, and play their role by acting as anti-inflammatory molecules, antioxidants, and wound-healing and angiogenesis accelerators. In COVID-19 BALB/c mouse models established by using the CoV-MHV-A59 virus, researchers found that thymosin drugs could balance the host immune response, and attenuate virus-induced inflammatory pathologic injury by inhibiting virus replication, and reducing the level of CRP and inflammatory mediators, thus promoting organ repair and increasing the survival rate of the mice infected with the MHV-A59 virus. These findings will provide a basic mechanism for further use of thymosin drugs in the treatment of COVID-19 and other coronal virus-associated diseases [15]. There are different reports about the protective value in COVID-19 patients, which we believe may be due to the different timing of using thymosin drugs. Thymosin drugs may have the ability of regulating immune responses to SARS-CoV-2 infection during the viremia phase by affecting the activities of T cells, NK cells and dendritic cells, regulating the generation of cytokines, and avoiding progression to cytokine storm, thus preventing evolution to severe COVID-19. By the time of progression of common COVID-19 infection to severe or critical pneumonia, inflammatory storm has occurred. The therapeutic effect of thymosin drugs used at this moment is minimal [16–18]. Thymosin drugs may have the ability of COVID-19 induced immune damage during the reactive phase of COVID-19 infection to prevent it from progressing to a cytokine storm [19]. In the SARS-CoV-2 pandemic setting, thymosin drugs can be used to prevent the occurrence of severe COVID-19 pneumonia in patients with relatively low autoimmune function such as elderly people, patients complicated with severe underlying diseases and advanced cancer patients [20–24].

## Conclusion

Thymalfasin therapy has shown excellent clinical efficacy in the treatment of COVID-19 pneumonia, it can reduce inflammatory reactions, promote the relief of COVID-19 pneumonia related symptoms such as fever and fatigue, facilitate effusion absorption, and accelerate COVID-19 pneumonia recovery. Thymalfasin can prevent progression of common COVID-19 infection to severe pneumonia via multiple immunity-enhancing and anti-inflammatory protective mechanisms.

## Data Availability

All datasets generated for this study are included in the article.
